# Nanoporous BNC network on Au(111) from a borazine-based arylalkyne

**DOI:** 10.1039/d5cc07416a

**Published:** 2026-03-10

**Authors:** Carolina M. Ibarra-Barreno, Martina Crosta, Joel Deyerling, Alida J. van Hunnik, Knud Seufert, Davide Bonifazi, Willi Auwärter, Petra Rudolf

**Affiliations:** a Zernike Institute for Advanced Materials, University of Groningen Groningen 9747 AG Netherlands p.rudolf@rug.nl; b Institute of Organic Chemistry, Faculty of Chemistry, University of Vienna Vienna 1090 Austria davide.bonifazi@univie.ac.at; c Physics Department E20, TUM School of Natural Sciences, Technical University of Munich Garching Germany wau@tum.de

## Abstract

On Au(111), an alkyne-terminated borazine derivative undergoes thermally induced cyclotrimerisation, forming nanoporous boron-nitrogen-carbon networks featuring regular BN-doped patterns, with borazine cores preserved during the reaction as characterised by scanning tunnelling microscopy (STM) and X-ray photoelectron spectroscopy (XPS).

## Abstract 2

On Au(111), an alkyne-terminated borazine derivative undergoes thermally induced cyclotrimerisation, forming nanoporous boron-nitrogen-carbon networks featuring regular BN-doped patterns.

## Graphical abstract

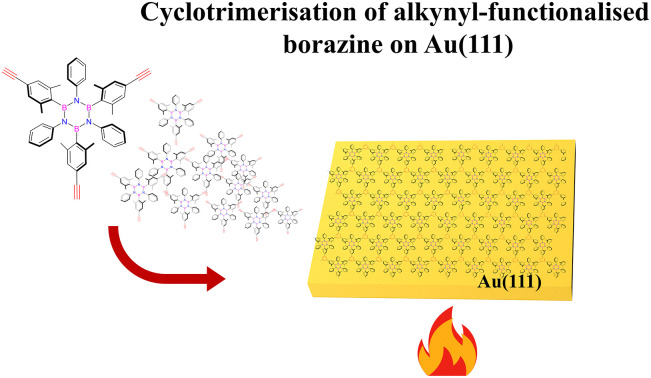

On-surface synthesis has emerged as a robust strategy for fabricating covalent organic frameworks (COFs) and functional two-dimensional (2D) networks.^1,2^ Typically initiated by thermal annealing, custom-designed precursor molecules adsorbed on metal substrates under ultra-high vacuum (UHV) conditions undergo coupling reactions.^3,4^ By stabilising and orienting the precursors, the metal surface not only enhances reaction selectivity compared to solution chemistry but also templates the formation of atomically precise 2D materials.^3^ Upon annealing at appropriate temperatures, intra- and intermolecular covalent bonds are formed, with common transformations including dehalogenative aryl–aryl coupling, cross-coupling, alkyne homocoupling and cyclotrimerisation.^1,2,5–7^ Specifically, previous reports have shown that terminal alkynes can undergo cyclotrimerisation on Au(111) under thermal activation to yield covalently linked porous 2D networks featuring aryl units.^8,9^ All-carbon aryl–alkyne precursors have further demonstrated to yield additional oligomeric structures and covalent architectures with different motifs.^8,10–13^ Thus, major challenges persist in identifying suitable molecular precursors for cyclotrimerisation reactions and in suppressing competing pathways and side reactions, which frequently hamper the formation of regular 2D covalent frameworks. In addition, although very rarely, the cyclotrimerisation strategy has been extended to explore heteroatom incorporation.^14,15^

Recent advances in the synthesis of heteroatom-doped graphene-like 2D materials have focused on incorporating non-metal atoms, such as N, B, P and S. These dopants can modify the graphenoid's density of states, thereby tailoring its properties for applications in electron transfer, catalysis and oxygen activation.^16–19^ In particular, B- and N-doping have emerged as a powerful strategy to engineer p- and n-type semiconductors, where the band gap and transport properties can be tuned by varying the BN-doping ratio.^20–22^ While B atoms contribute to p-type conductivity, N atoms provide electron-donating features that enhance n-type behaviour.^19,23,24^ Although this approach is now well established at the molecular level,^25–28^ a central challenge remains the controlled and periodic incorporation of BN bonds into 2D materials, avoiding the segregation of thermodynamically favoured boron nitride nanodomains.^29^ If realised, such materials could expand the application space of BN-doped 2D systems in catalysis, gas storage and optoelectronics.^26–28^

On-surface chemistry provides an attractive route to address this challenge. Borazine-based molecular modules, featuring a B_3_N_3_ core, have been explored as precursors for engineering heteroatom-doped porous carbon-based 2D networks.^30^ Depending on their design, borazines can be decorated with aryl substituents (inner shell) at the B and N positions and further functionalised with terminal reactive groups (outer shell), enabling the fabrication of BNC materials (Fig. 1a).^31^ Importantly, when fabrication protocols are compatible with the thermal stability of the B_3_N_3_ core, a wide variety of BN-doped 2D materials can be achieved by exploiting the chemical diversity of the inner-shell substituent.

**Fig. 1:**
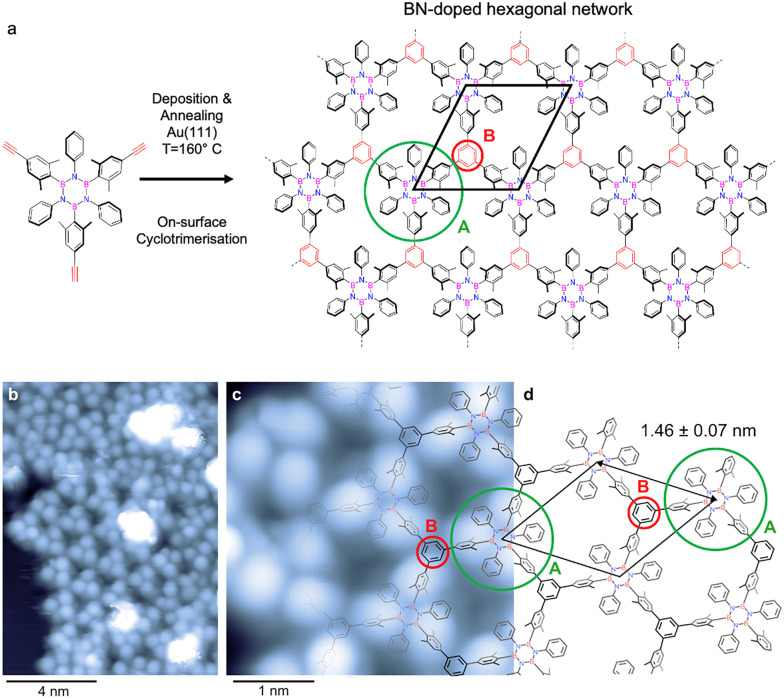
(a) Scheme of alkyne-terminated borazine precursor (left) and hypothesised 2D porous BN-doped hexagonal network (right) formed *via* thermal on-surface cyclotrimerisation as investigated in this work. (b)–(d): STM characterisation after depositing the alkyne-terminated borazine precursor onto Au(111) held at 160 °C. (b) STM image showing an island of molecules mainly constituted by a hexagonal molecular framework. (2.0 V, 10 pA) (c) High-resolution STM image of the hexagonal framework. (−0.8 V, 10 pA) (d) Schematic model of the network. The average distance between B_3_N_3_ cores extracted from STM images is indicated and selected sites representing the sublattices A and B are marked by coloured circles (compare (a)).

Designing precursors that covalently link into ordered networks not only prevents molecular desorption from the surface but also constitutes a crucial step toward the controlled fabrication of borazine-doped graphene. Building on this concept, in this work, we explore the use of an alkyne-terminated borazine derivative, *N,N*′*,N*″*-triphenyl-B,B*′*,B*″*-tri(2,6-dimethyl-4-ethynylphenyl)borazine* (Fig. 1a), as a molecular precursor to fabricate 2D nanoporous BNC networks on Au(111). Upon annealing, the terminal alkynes are envisaged to undergo on-surface intermolecular cyclotrimerisation,^8,10,11^ with each newly formed benzene ring covalently bridging three borazine modules. This approach provides a direct route to extend the cyclotrimerisation strategy, previously demonstrated for all-carbon aryl–alkyne systems, toward the bottom-up synthesis of heteroatom-doped 2D frameworks.^3,8,10,32^ The borazine-based molecular precursor investigated in this work was prepared following the procedure reported by us (see SI).^28,33,34^

Low temperature scanning tunneling microscopy (LT-STM) experiments were performed in a custom-built UHV chamber housing a commercial low temperature STM (CreaTec) operated at ∼8 K (−265 °C) and the images were obtained in constant-current mode. Prior to each experiment, the Au(111) surface was cleaned by several cycles of sputtering (Ar^+^ ions, *E*_kin_ = 1 keV) and annealing at 450 °C. After the substrate cleaning, the alkyne-terminated borazine was sublimed from a commercial molecular beam epitaxy setup (OMBE, Dodecon) kept at 150–160 °C onto the Au(111) held at 160 °C for 60 min, resulting in a submonolayer coverage of molecules.

In a different UHV system, the X-ray photoelectron spectroscopy (XPS) data were collected *in situ* after deposition and annealing using a Scienta R4000 spectrometer with a monochromatic Al *K*_*α*_ X-ray source (*hv* = 1486.6 eV) and a hemispherical electron analyser equipped with a multichannel detector system and a CCD camera. The incident light was at a 45° angle from the surface normal, and photoelectrons were detected in normal emission; the base pressure within the spectrometer was approximately 9 × 10^−10^ mbar. The spectra were fitted with the minimum number of peaks consistent with the molecular structure on the surface. Before each XPS experiment, the Au(111) surface was cleaned by several cycles of sputtering (Ar^+^ ions, *E*_kin_ = 1 keV) and annealing in UHV (10^−9^ mbar) at 420 °C. After cleaning, the alkyne-terminated borazine was sublimed from a custom-made molecular evaporator heated to 140 °C onto the Au(111) substrate held at room temperature (*rt*) for 60 min to ensure uniform coverage. After sublimation, the sample was annealed at 160 °C for 60 min.

Fig. 1 shows STM images of the alkyne-terminated borazine adsorbed on Au(111), held at 160 °C during deposition. This temperature was expected to trigger the cyclotrimerisation reaction.^8^ The overview image (Fig. 1b) reveals a molecular island, including a porous array (centre) and denser assemblies at the periphery (top left). The latter include unreacted monomers, as concluded from the molecular dimensions. The porous array dominates the image (∼65%), revealing a characteristic hexagonal network with two distinct sublattices: a B_3_N_3_ core surrounded by aromatic rings (core and inner shell) (sublattice A) and the benzene ring formed during the cyclotrimerisation reaction (sublattice B). If we focus on a B_3_N_3_ core and its inner shell, three pronounced protrusions are visible and can be assigned to the B-linked dimethylaryl moieties.

Fig. 1c provides a more detailed view of the regular hexagonal framework (as shown in Fig. 1d with the structural representation of the 2D network). The B-linked dimethylaryl groups dominate the submolecular contrast, consistent with STM data of individual functionalised borazines with dimethylaryl moieties reported earlier for different coinage metal supports.^19^ Additionally, one can distinguish contributions from the terminal phenyl moieties and the central B_3_N_3_ units (see Fig. 1c).

The porous array is described by a rhombic unit cell including one B_3_N_3_ core with inner shell and one additional, linking benzene ring as building blocks (Fig. 1c and d). Based on the STM data, the unit cell's side length equals 1.46 ± 0.07 nm, which is consistent with the distance expected for a covalent network structure, and represents the borazine-borazine separation. The irregular protrusions with considerable apparent height (white contrast in Fig. 1b) correspond to adsorbates on top of the molecular layer.

A closer inspection of high-resolution STM data shows a chiral character (Fig. 2a and b) of the borazine units constituting the network. The three B-linked dimethylaryl moieties per BN subunit (B_3_N_3_ core with inner shell) are tilted out of plane in the same direction, inducing a non-planar structure (Fig. 2a). The two equivalent configurations result in either a clockwise or counterclockwise appearance of the unit (highlighted by the arrows in Fig. 2a and b).

**Fig. 2:**
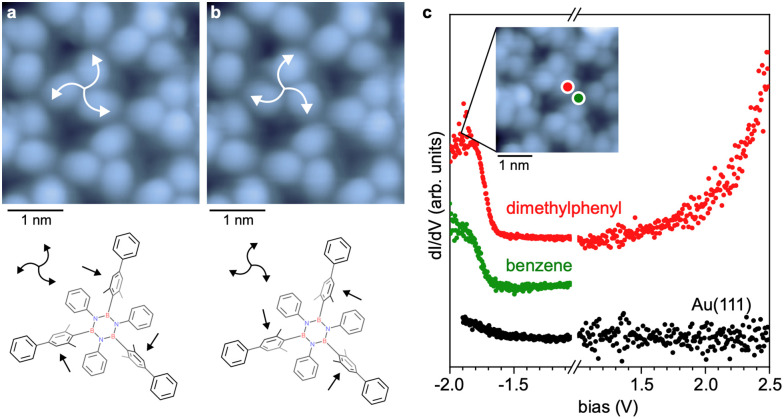
Structural and electronic properties of the subunit in the hexagonal BNC network. (a) A high-resolution STM image of the hexagonal network reveals the central molecule's chirality (highlighted by curved arrows). (−1.0 V, 10 pA). (b) STM image of the same area where the chirality of the central molecule was switched. (−1.0 V, 10 pA). The bottom panels in (a) and (b) present a sketch of the configurations of submolecular units, with straight arrows pointing to the tilted dimethylphenyl moieties. (c) d*I*/d*V* spectroscopy of the hexagonal BNC network. Spectra are vertically offset for clarity. The inset shows an STM image of the hexagonal BNC network measured at the first occupied state (−1.9 V, 10 pA). The coloured dots mark the positions of the spectra.

This rotational degree of freedom and molecular flexibility can lead to the structural order of the network being underestimated in STM images (*e.g*. Fig. 1b). Under perturbative scanning conditions (*e.g.* −1.0 V, 10 pA), the chirality can be reversibly switched (Fig. 2b). Significantly, this switching of the submolecular units does not alter the overall network structure, which aligns with the stability expected for a covalent architecture.

Differential conductance (d*I*/d*V*) spectra recorded on dimethylphenyl positions adjacent to the B_3_N_3_ cores show contributions from both occupied and unoccupied electronic states (Fig. 2c). A step-like feature occurs at ∼ −1.8 V, and a more continuous increase in the d*I*/d*V* signal is observed above 2.0 V, indicating a sizeable electronic gap. The onset at about −1.8 V persists on the phenyl nodes of the network, signalling some delocalisation, which is expected from the extended π-conjugation. Nonetheless, we refrain from assigning this spectral fingerprint to the valence band of the network, as a more local character, given by the highest occupied molecular orbital, cannot be ruled out. Density functional theory^35^ calculations on a trimeric model reveal occupied electronic states on both the dimethylphenyl moieties and the benzene nodes (see Fig. S9). A bias-dependent STM image series is presented in Fig. S10, revealing subtle contrast variations consistent with the d*I*/d*V* signatures and confirming that the STM contrast is dominated by “topographic” features emerging from the tilted dimethylaryl moieties.

Beyond inherently local STM measurements, the BNC network was also characterised by XPS to provide information about the chemical environment after the thermal treatment. The XPS spectra confirm that the molecular precursor was successfully deposited on Au(111) (Fig. S11) and could be processed to yield the BNC nanoporous network without degradation. Fig. 3 shows the spectra of the C 1s, N 1s and B 1s core level regions, collected after alkyne-terminated borazine deposition at *rt* and after annealing at 160 °C. After deposition at *rt*, the C 1s spectrum (Fig. 3, left panel, bottom) can be deconvoluted into three components. The main component at a binding energy (BE) of 284.4 eV corresponds to carbon–carbon bonds (C–C, C=C and C≡C); unfortunately, our experimental resolution does not allow to identify the different bonds as distinct components. Additional contributions at BEs of 283.7 and 285.3 eV stem from C–B and C–N bonds, respectively.^27–29^ The N 1s spectrum (Fig. 3, middle panel, bottom) consists of a single component at a BE of 398.7 eV, which is attributed to C–N–B_2_ (nitrogen bound to boron in the B_3_N_3_ core).^27–29,36^ The B 1s spectrum (Fig. 3, right panel, bottom) peaks at 190.0 eV, consistent with the C–B–N_2_ structure of the B_3_N_3_ core.^27–29,36^ The composition of the adsorbed layer as deduced from the XPS intensities was found to be C = 87.8 ± 0.5 at%, N = 6.7 ± 1.1 at% and B = 5.5 ± 2.1 at%, which agrees with the stoichiometry (C = 88.8 at%, N = 5.6 at%, B = 5.6 at%) of the molecule, and no other elements were found. The absence of additional components in the N 1s and B 1s spectra, combined with the agreement between the experimental atomic percentages and the molecular stoichiometry, indicates that the B_3_N_3_ core remained intact during sublimation and surface adsorption.

**Fig. 3:**
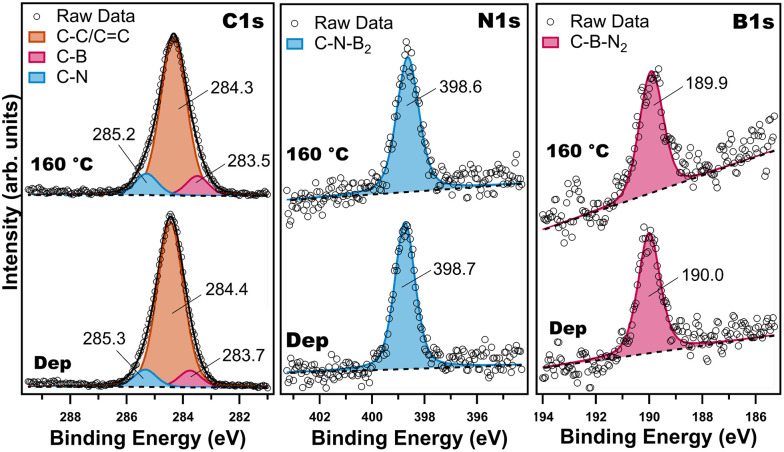
XPS spectra of the C 1s (left), N 1s (middle) and B 1s (right) core-level regions of alkyne-terminated borazine derivative on Au(111) after deposition at *rt* and after annealing at 160 °C. The corresponding fits are also shown.

To form the BNC network, the Au(111) crystal was annealed at 160 °C; XPS spectra were then collected after the thermal treatment. As shown in Fig. 3, all core-level spectra shifted slightly 0.1 eV towards lower BEs, and the full width half-maximum (FWHM) increased by 0.1 eV after annealing, pointing to a slight change in the chemical environment. The C 1s spectrum (Fig. 3, left panel, top) can still be deconvoluted into three components peaked at 283.5, 284.3 and 285.2 eV, corresponding to C–B, C–C/C=C and C–N bonds, respectively. The N 1s spectrum (Fig. 3, middle panel, top) continues to exhibit a single component at 398.6 eV, corresponding to C–N–B_2_ (B_3_N_3_ core)^27,28^ and also the B 1s spectrum (Fig. 3, right panel, top) still shows only one peak at 189.9 eV, consistent with the C–B–N_2_ structure of the B_3_N_3_ core.^27,28^ Taken together, these results confirm that the borazine rings retained their integrity after annealing. Subsequently, we also checked if the BNC network was also stable at higher temperatures, such as 250 °C. This thermal treatment modified the atomic percentages of C, N and B (details can be found in Fig. S12 and Table S1), however the covalent network chemical bonding remained on the surface (Fig. S12), suggesting that the molecular precursors do not desorb after the cyclotrimerisation.

Since the molecules were directly deposited on a hot surface in the STM experiment, XPS spectra were also collected on the LT-STM sample (experimental details can be found in the SI). Fig. S13 shows the spectra of C 1s, N 1s and B 1s core level regions, collected after deposition of the borazine on a hot surface at 160 °C. The C 1s (Fig. S13, left panel) spectrum can be deconvoluted into three components, peaked at the BEs of 283.5, 284.2 and 285.1 eV, corresponding to C–B, C–C/C=C and C–N bonds, respectively. The N 1s spectrum (Fig. S13, middle panel) consists of a single component at a BE of 398.7 eV, which is attributed to C–N–B_2_ motif, and the B 1s spectrum (Fig. S13, right panel) of a single component peaked at 190.0 eV, attributed to C–B–N_2_. This confirms that annealing after deposition at *rt* and hot deposition result in the same chemical environment.

In summary, in this paper, we have reported the on-surface synthesis of a nanoporous covalent network of functionalised borazine units *via* cyclotrimerisation of a borazine precursor bearing three alkynyl moieties on Au(111) at 160 °C. XPS and LT-STM investigations suggested the formation of a hexagonal network (1.46 nm periodicity) with the hexaryl-substituted borazine centres expressing chirality. Interestingly, their chirality can be switched reversibly by STM. Since this method preserves the B_3_N_3_ core within the network, it offers a promising pathway towards the rational design of structurally tailored 2D BNC materials.

C. M. Ibarra-Barreno: conceptualisation, methodology, investigation, formal analysis, visualisation, writing – original draft preparation, writing – reviewing & editing. M. Crosta: conceptualisation, methodology, investigation, formal analysis, visualisation, writing – original draft preparation, writing – reviewing & editing. J. Deyerling: conceptualisation, methodology, investigation, formal analysis, visualisation, investigation, writing – original draft preparation, writing – reviewing & editing. A. van Hunnik: data curation, investigation. K Seufert: methodology, investigation, writing – reviewing & editing. D. Bonifazi: conceptualisation, validation, supervision, writing – original draft preparation, writing – reviewing & editing, funding acquisition, supervision. W. Auwärter: conceptualisation, validation, writing – original draft preparation, writing – reviewing & editing, funding acquisition, supervision. P. Rudolf: conceptualisation, validation, writing – original draft preparation, writing – reviewing & editing, funding acquisition, supervision.

## Conflicts of interest

There are no conflicts to declare.

## Supplementary Material

CC-062-D5CC07416A-s001

## Data Availability

Supplementary information (SI): general remarks on precursor synthesis; synthetic procedures and spectral data; NMR spectra; gas phase DFT calculations; additional XPS and STM characterisation data of the nanoporous network. See DOI: https://doi.org/10.1039/d5cc07416a.
