# Supplementary material for: Nanoporous BNC network on Au(111) from a borazine-based arylalkyne
Source: Chem Commun (Camb). 2026 Mar 10;62(26):6945–9. doi: 10.1039/d5cc07416a (PMC12997529; doi:10.1039/d5cc07416a)
Supplement: CC-062-D5CC07416A-s001 [file CC-062-D5CC07416A-s001.pdf]

Supplementary Information (SI) for ChemComm.
This journal is © The Royal Society of Chemistry 2026

Supplementary Information

Nanoporous BNC network on Au(111) from a borazine-based arylalkyne

Carolina M. Ibarra-Barreno,[‡a] Martina Crosta,[‡b] Joel Deyerling,[‡c] Alida van Hunnik,[a] Knud Seufert,[c] Davide Bonifazi,[*b] Willi Auwärter,[*c] Petra Rudolf [*a]

[a] *Zernike Institute for Advanced Materials, University of Groningen, Nijenborgh 3, 9747 AG, Groningen, The Netherlands*

[b] *Institute of Organic Chemistry, Faculty of Chemistry, University of Vienna, Währinger Strasse 38, 1090 Vienna, Austria*

[c] *Physics Department E20, TUM School of Natural Sciences, Technical University of Munich, James-Franck-Str. 1, 85748 Garching, Germany*

*Corresponding authors: p.rudolf@rug.nl; wau@tum.de; davide.bonifazi@univie.ac.at

Table of contents

S1 Synthesis of precursors: General remarks 2

S1.1 Instrumentation 2

S1.2 Materials and methods 3

S2 Synthetic procedures and spectral data 3

S2.1 Synthesis of ((4-bromo-3,5-dimethylphenyl)ethynyl)trimethylsilane 1 3

S2.2 Synthesis of *N*,*N*',*N*''-triphenyl-*B*,*B*',*B*''-tri(2,6-dimethyl-4-((trimethylsilyl)ethynyl)phenyl)borazine 2 .. 4

S2.3 Synthesis of *N*,*N*',*N*''-triphenyl-*B*,*B*',*B*''-tri(2,6-dimethyl-4-ethynylphenyl)borazine 3 5

S3 NMR spectra 6

S3.1 Characterization of 1 6

S3.2 Characterization of 2 7

S3.3 Characterization of 3 8

S4 Gas-phase DFT calculations 10

S5 Characterization of the nanoporous covalent network 19

S6 References 22

S1 Synthesis of precursors: General remarks

S1.1 Instrumentation

Thin layer chromatography (TLC) was conducted on pre-coated aluminium sheets with 0.20 mm Merck Millipore Silica gel 60 with fluorescent indicator F254.

Column chromatography was carried out using *Merck Gerduran* silica gel 60 (particle size 40-63 μm).

Nuclear magnetic resonance (NMR) ^{1}H, ^{13}C, and ^{11}B spectra were obtained on a Bruker spectrometer AV III 600 or AV NEO 400 at the *NMR centre* of the *University of Vienna*. All spectra were obtained at room temperature (rt). Carbon spectra were acquired with a complete decoupling for the proton. Boron spectra were measured in quartz NMR tubes. Proton and carbon chemical shifts are reported in parts per million (ppm, δ scale) according to tetramethylsilane ($\delta_H = \delta_C = 0$ ppm) using the solvent residual signal (*) as an internal reference ($CDCl_3$: $\delta_H = 7.26$ ppm). Boron chemical shifts are reported in ppm, referenced to the external standard boron signal of $BF_3{\cdot}Et_2O$ ($\delta_B = 0$ ppm). Coupling constants (*J*) are given in Hz. Resonance multiplicity is described as s (singlet) and m (multiplet).

High-resolution mass spectrometry ***(HRMS)*** analyses were performed by the *Mass Spectrometry Centre* at the *University of Vienna*. GC mass spectra were measured on an Agilent 7200B GC/Q-TOF mass spectrometer. MALDI-MS spectra were recorded on a Bruker Autoflex Speed MALDI-timsTOF (matrix: 2-[(2E)-3-(4-tert-butylphenyl)-2-methylprop-2-enylidene]malononitrile (DCTB)) mass spectrometer.

S1.2 Materials and methods

Chemicals were purchased from *Sigma Aldrich*, *Acros Organics*, *TCI*, *ABCR*, *Alfa Aesar, Fluorochem, Thermo Fisher Scientific* and *BLDpharm* and used as received. Solvents were purchased from *Sigma Aldrich*, while deuterated solvents from *Eurisotop*. THF was distilled from sodium-benzophenone, toluene was refluxed over calcium hydride. Aniline was distilled from calcium hydride. Anhydrous conditions were achieved by drying glassware in oven at 120 °C for at least 12 h and by flaming the reaction vessels with a heat gun under vacuum and purging with argon. The inert atmosphere was maintained using argon-filled balloons equipped with a syringe and needle that was used to penetrate the silicon septa used to close the flask's necks. Alternative to the use of Schlenk line techniques, inert conditions were achieved by using an argon-filled MBraun LabStar glove box when stated. Addition of liquid reagents was performed using argon-purged plastic or glass syringes. Degassing of solutions was performed by *freeze-pump-thaw* procedure: solutions were frozen in liquid nitrogen and kept under vacuum for 10–15 min before thawing. Low temperature baths were prepared using different solvent mixtures depending on the desired temperature: -84°C with liquid N_2/EtOAc, and 0 °C with ice/water.

S2 Synthetic procedures and spectral data

S2.1 Synthesis of ((4-bromo-3,5-dimethylphenyl)ethynyl)trimethylsilane 1

Prepared according to literature procedure.[1]

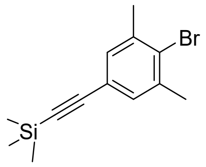

In a 150 mL Schlenk tube, to a solution of 2-bromo-5-iodo-*m*-xylene (2.99 g, 9.60 mmol) in diisopropylamine (60 mL), $[PdCl_2(PPh_3)_2]$ (202 mg, 0.288 mmol) and CuI (110 mg, 0.576 mmol) were added. The mixture was subjected to three *freeze-pump-thaw* cycles and

trimethylsilylacetylene (1.64 mL, 11.5 mmol) was added. The mixture was stirred at room temperature for 16 h, then diluted with water (50 mL) and extracted with CH_2Cl_2 (3 x 80 mL). The combined organic layers were dried over Na_2SO_4 and the solvents removed under reduced pressure. The residue was purified by short silica gel plug filtration (heptane), affording **1** as a yellow oil (2.78 g, quantitative yield). The product was further purified by distillation over CaH_2 (0.04 mbar, 105 °C) for subsequent lithiation reaction. Spectral properties are in agreement with those reported in the literature.[1]

^{1}H NMR (400 MHz, $CDCl_3$) δ 7.19 (s, 2H), 2.38 (s, 6H), 0.27 (s, 9H). ^{13}C NMR (101 MHz, $CDCl_3$) δ 138.4, 131.4, 128.3, 121.6, 104.6, 94.6, 23.8, 0.09. GC-HRMS $[M]^+$ calc. for $[C_{13}H_{17}BrSi]^+$: 282.0257, found: 282.0251.

S2.2 Synthesis of *N*,*N'*,*N''*-triphenyl-*B*,*B'*,*B''*-tri(2,6-dimethyl-4-((trimethylsilyl)ethynyl)phenyl)borazine 2

Prepared according to literature procedure.[1]

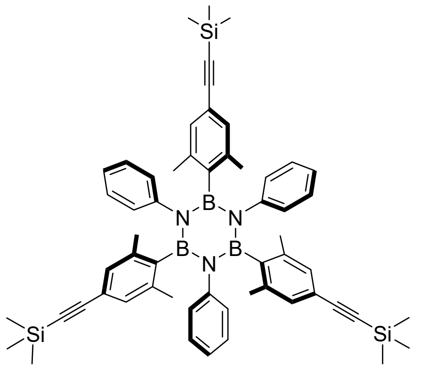

All manipulations of solids and solvents were done in an argon-filled glove box. In a 100 mL Schlenk tube, a solution of BCl_3 (1 M in heptane, 13 mL, 13 mmol) was added dropwise to a solution of distilled aniline (0.54 mL, 5.9 mmol) in dry toluene (10 mL) at 0 °C. The white suspension was refluxed for 20 h. The solution was allowed to reach room temperature, and all volatiles were removed *in vacuo*, then the mixture was dissolved in dry THF (10 mL). Parallelly, in a 100 mL Schlenk tube, *t*BuLi (2.3 M in heptane, 6.5 mL, 14.9 mmol) was added dropwise over 10 min to a solution of **1** (2.0 g, 7.1 mmol) in dry THF (31 mL) at -84 °C. The solution was stirred for 10 min at 0 °C. The chloro-borazole solution (at 0 °C) was cannulated dropwise to the aryl lithium solution (at 0 °C). The solution was stirred at 0 °C for 10 min and at room temperature for 16 h. The reaction mixture was diluted with EtOAc (100 mL), washed with water (2 x 100 mL) and brine (100 mL). The organic layer was dried over Na_2SO_4 and

concentrated under reduced pressure. The residue was purified by precipitation in MeOH and silica gel plug filtration (CH_2Cl_2) affording **2** as a white solid (466 mg, 26 % yield). Spectral properties are in agreement with those reported in the literature.[1]

^{1}H NMR (400 MHz, $CDCl_3$) δ 6.83–6.60 (m, 21H), 2.24 (s, 18H), 0.15 (s, 27H). ^{13}C NMR (151 MHz, $CDCl_3$) δ 145.4, 137.5, 129.0, 127.2, 126.5, 125.0, 121.1, 106.0, 92.8, 22.9, -0.04 (one signal is missing due to ^{11}B-induced quadrupolar relaxation). ^{11}B NMR (193 MHz, $CDCl_3$) δ 38.0. MALDI-HRMS [M]$^+$ calc. for $[C_{57}H_{66}B_3N_3Si_3]^+$: 909.4865, found: 909.4858.

S2.3 Synthesis of *N*,*N'*,*N''*-triphenyl-*B*,*B'*,*B''*-tri(2,6-dimethyl-4-ethynylphenyl)borazine 3

Prepared according to literature procedure.[1]

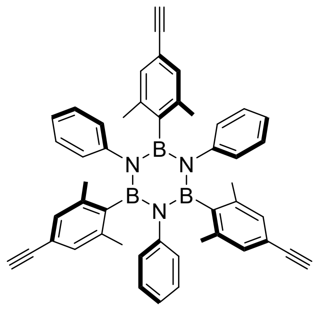

In a 10 mL round-bottom flask, TBAF (1 M solution in THF, 0.37 mL, 0.37 mmol) was added dropwise to a solution of **2** (100 mg, 0.11 mmol) in THF (2.6 mL) at 0 °C. The reaction mixture was stirred at room temperature for 1 h, then diluted with brine (25 mL) and extracted with CH_2Cl_2 (3 x 40 mL). The combined organic layers were dried over Na_2SO_4 and the solvents removed under reduced pressure. The residue was purified by silica gel column chromatography (heptane/CH_2Cl_2 9:1 to 7:3) affording **3** as a white solid (57 mg, 75 % yield). Spectral properties are in agreement with those reported in the literature.[1]

^{1}H NMR (600 MHz, $CDCl_3$) δ 6.86–6.73 (m, 15H), 6.70 (s, 6H), 2.87 (s, 3H), 2.26 (s, 18H). ^{13}C NMR (151 MHz, $CDCl_3$) δ 145.4, 137.8, 129.3, 127.3, 126.6, 125.2, 120.4, 84.5, 76.1, 23.0 (one signal is missing due to ^{11}B-induced quadrupolar relaxation). ^{11}B NMR (193 MHz, $CDCl_3$) δ 36.2. MALDI-HRMS [M]$^+$ calc. for $[C_{48}H_{42}B_3N_3]^+$: 693.3674, found: 693.3679.

S3 NMR spectra

S3.1 Characterization of 1

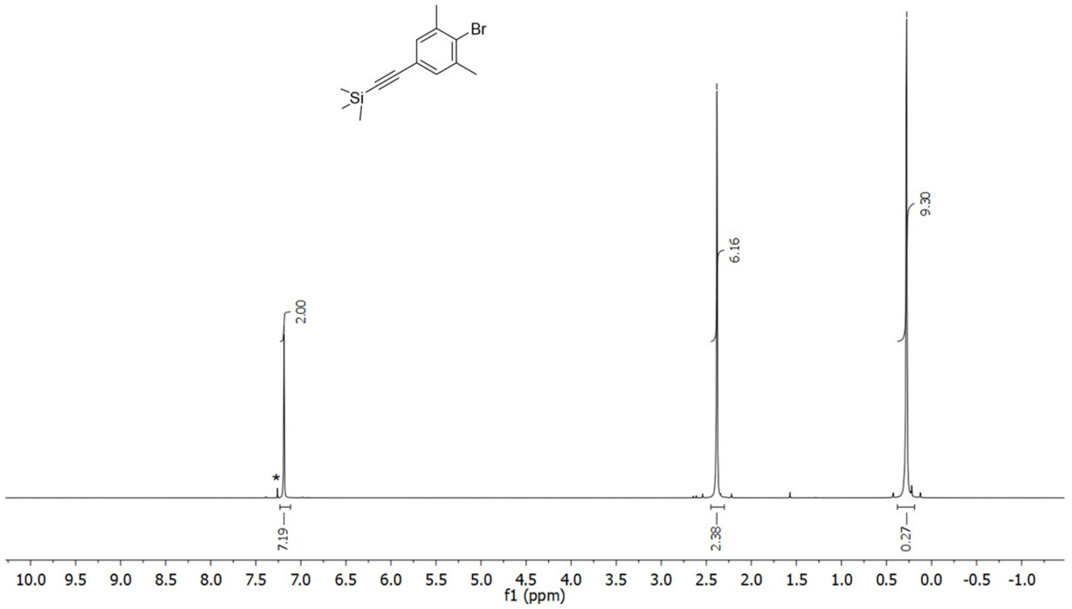

Figure S1.^{1}H NMR (400 MHz, $CDCl_3$) spectrum.

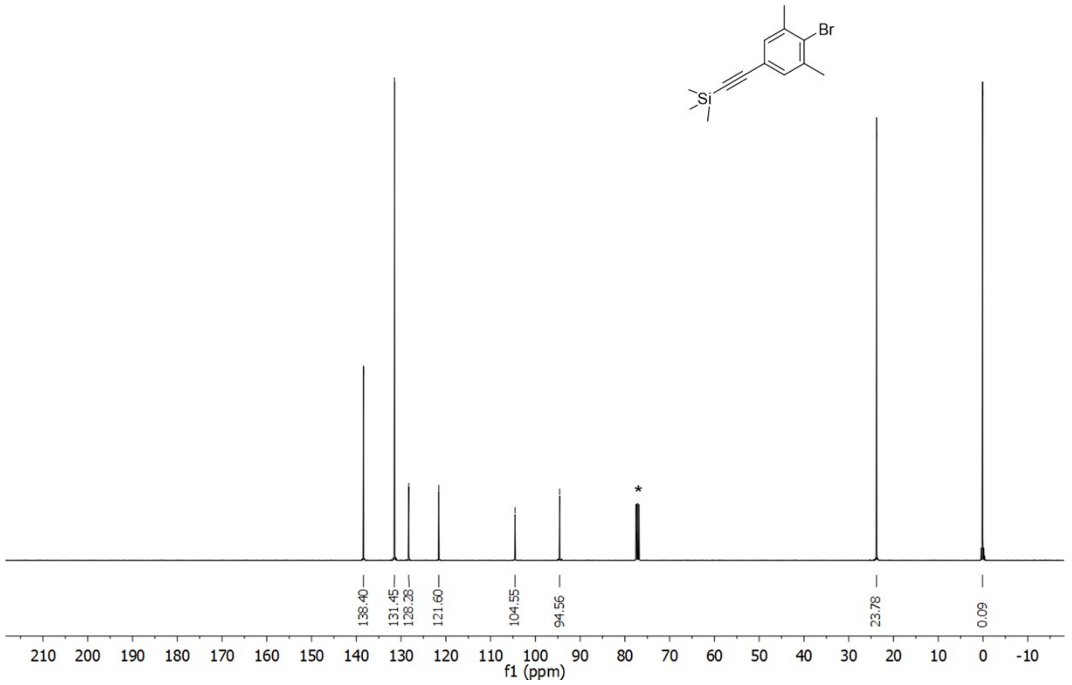

Figure S2.^{13}C NMR (101 MHz, $CDCl_3$) spectrum.

S3.2 Characterization of 2

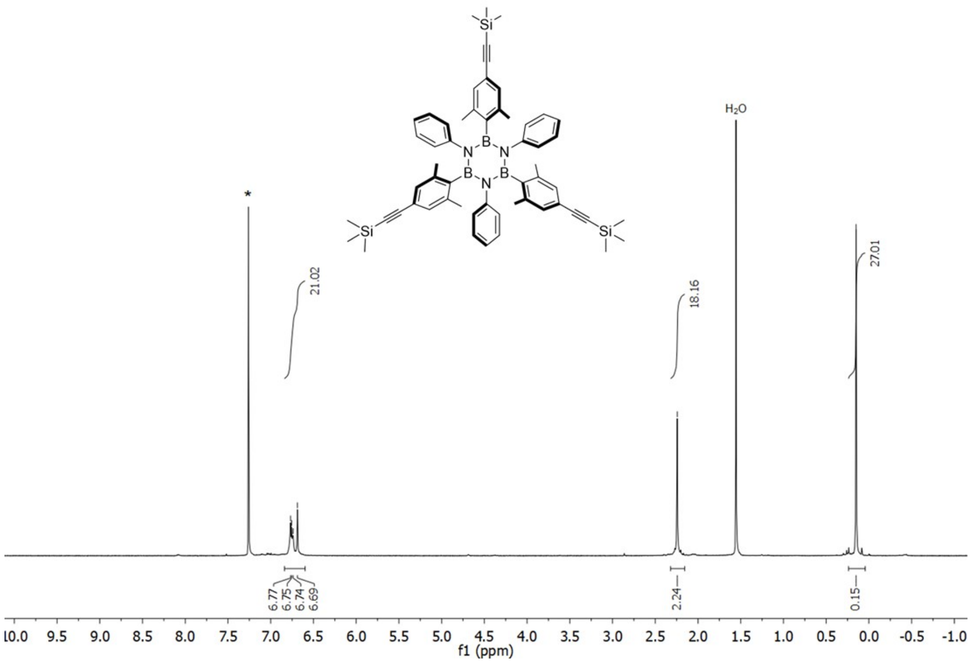

Figure S3. ^{1}H NMR (400 MHz, $CDCl_3$) spectrum.

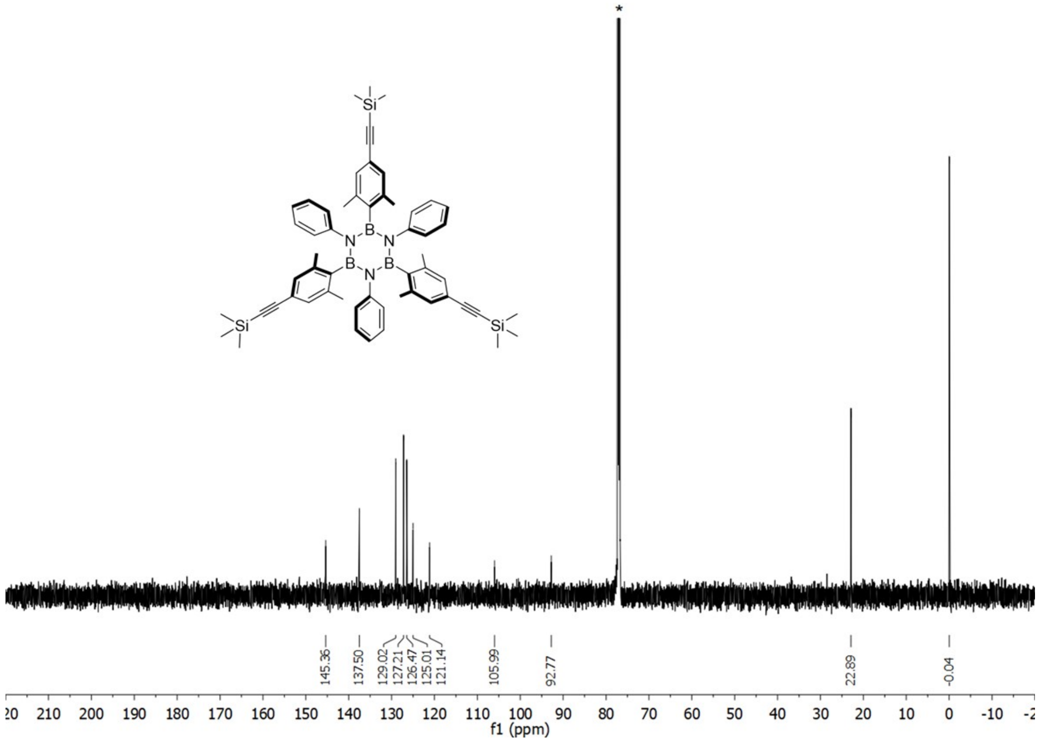

Figure S4. ^{13}C NMR (151 MHz, $CDCl_3$) spectrum.

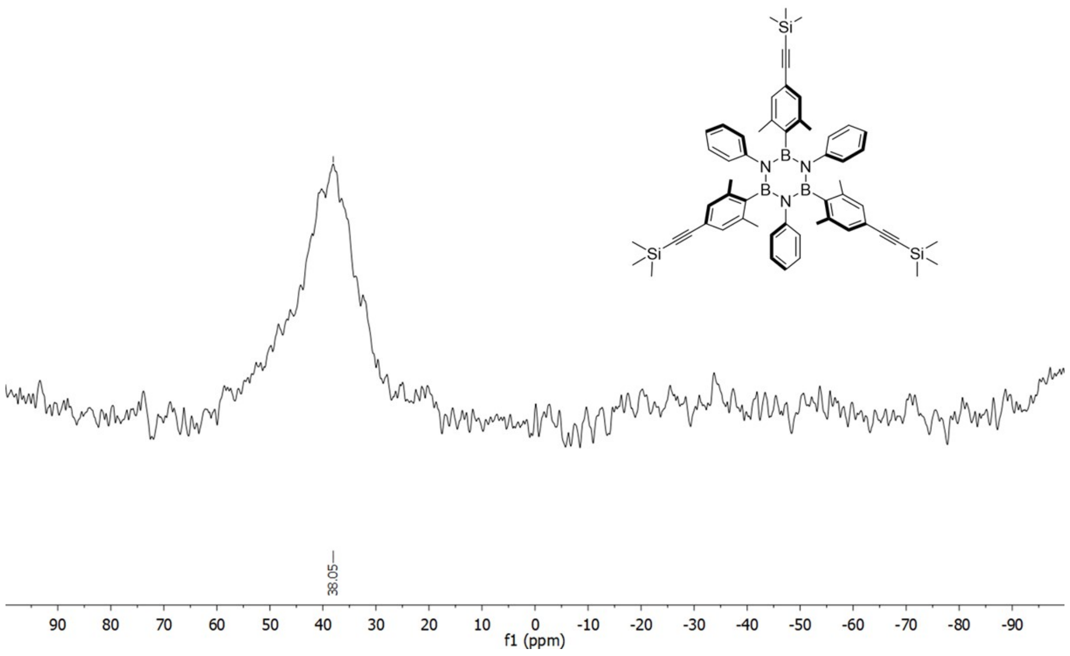

Figure S5. ^{11}B NMR (193 MHz, $CDCl_3$) spectrum.

S3.3 Characterization of 3

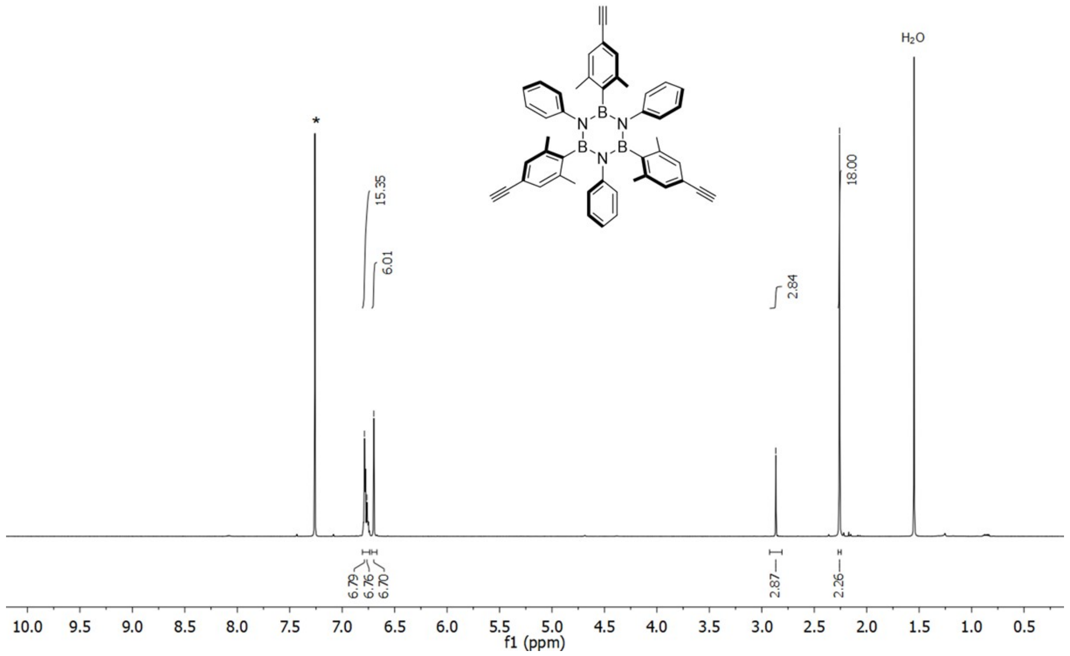

Figure S6. ^{1}H NMR (600 MHz, $CDCl_3$) spectrum.

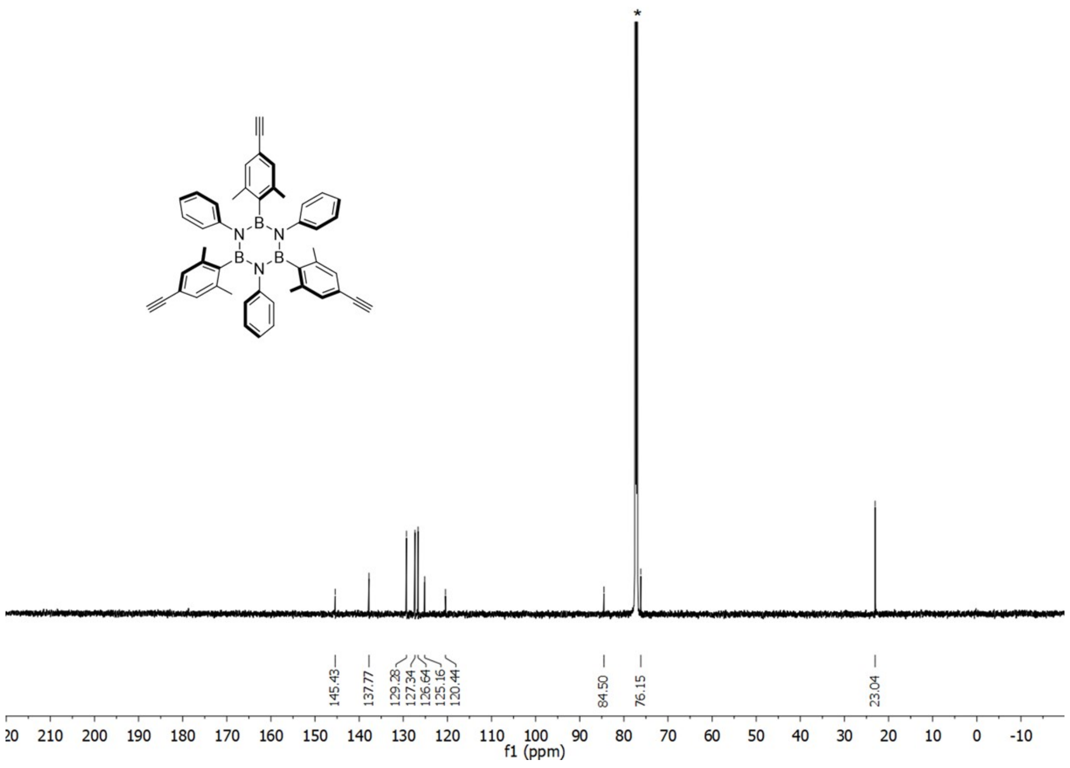

Figure S7. ^{13}C NMR (151 MHz, $CDCl_3$) spectrum.

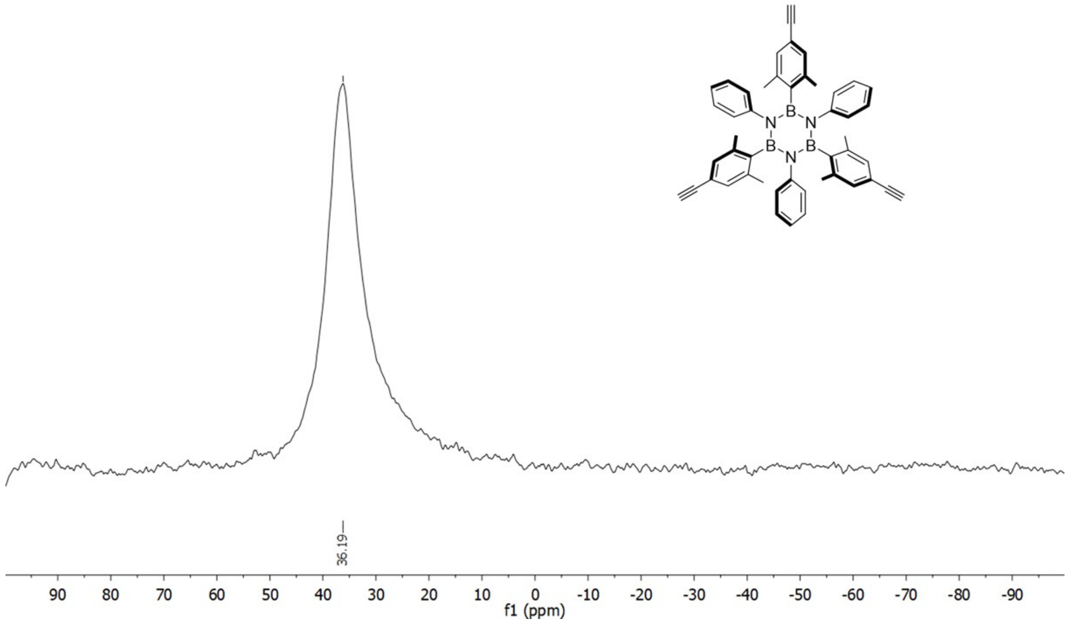

Figure S8. ^{11}B NMR (193 MHz, $CDCl_3$) spectrum.

S4 Gas-phase DFT calculations

Restricted Kohn-Sham DFT calculations of the trimer in gas-phase were conducted with ORCA (6.0.0).[2] For the geometry optimization and single-point energy calculations the B3LYP functional with the SVP basis-set and def2/J auxiliary basis-set was used in combination with D3 dispersion correction. Due to the large size of the trimer, we limited the calculations to require "LooseOPT" for the geometry and "NormalSCF" for the SCF. The optimization was run with the grid "defgrid3" and in cartesian coordinates "COPT".

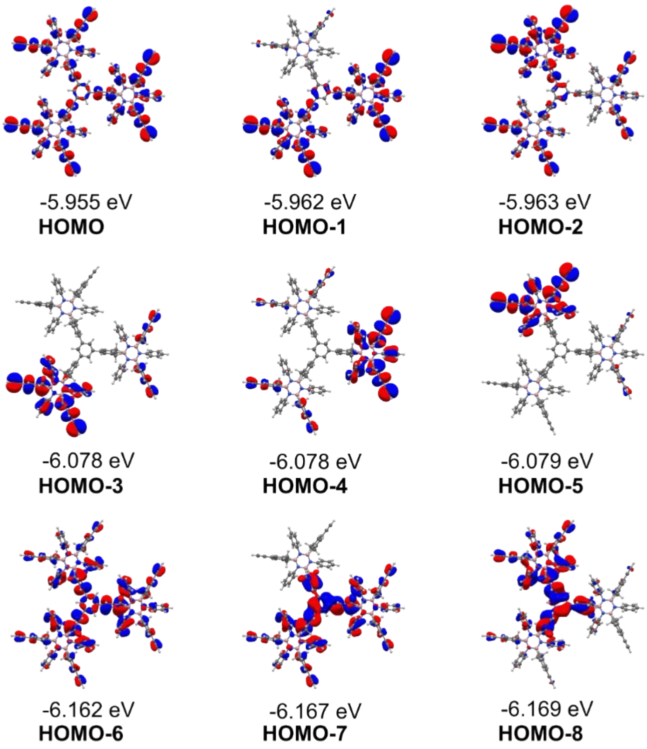

Figure S9. Overview of the occupied gas-phase DFT calculated orbitals of the molecular trimer. The isosurfaces are set to 0.01 [a.u.].

Structure of the relaxed trimer in cartesian coordinates [Å]:

B	-1.35260927051924	21.59414106243853	0.10816839486428
N	-0.45727530910039	22.72656443618584	0.13808965237681
B	0.97880130054047	22.57144856818365	0.15111175932502
N	1.50988649895475	21.22853802731270	0.14326879652649
N	-0.76778756732095	20.27499397522939	0.08523069319075
C	2.93291274645302	21.04629540624024	0.17170229753116

C	-1.63589829513901	19.13272997029898	0.04080449819417
C	-1.01207950879146	24.04982813933374	0.15703752664914
C	3.71769184069837	21.41088488911570	-0.92665879502424
C	5.10224647201412	21.22954137309576	-0.89792312178192
C	5.71557126288061	20.67967112367888	0.23183850905335
C	4.93442471260872	20.31269061874437	1.33062877870330
C	3.55001231877525	20.49640382720862	1.30122276873392
C	-2.42975297482369	18.80272539550935	1.14389397476173
C	-3.26981479506963	17.68799637845289	1.10184047696734
C	-3.32473096479330	16.89232053730780	-0.04649150884301
C	-2.53102586421282	17.21823122145424	-1.15033639118003
C	-1.69266654915831	18.33494865214739	-1.10635408978892
C	-0.90799723381242	24.84391687715129	1.30433536592152
C	-1.44740056922965	26.13229114248628	1.32397134755838
C	-2.09728634505470	26.63915036817394	0.19597410049014
C	1.93902277460893	23.83159933594290	0.16868295057427
C	2.53805539818826	24.26528584326995	1.37162234141385
C	3.38650084164909	25.37517153809789	1.37394620908904
C	3.67547321262856	26.07045353855092	0.18788224104836
C	3.08224438572013	25.63197206702566	-1.00754244087121
C	2.21839830546719	24.53411914536251	-1.02408854820426
C	-2.92505241048719	21.79092697626134	0.10377835817003
C	-3.61631220400296	22.05385742614848	1.30654784900493
C	-5.00535902728387	22.20329254955072	1.30026154301414
C	-5.73951554937941	22.11355901392631	0.10593532383278
C	-5.04610863724297	21.86338870987362	-1.09014749003244
C	-3.65944805099580	21.69460680827904	-1.09849251162179
B	0.66087369365222	20.05930684371860	0.10634160444580
C	1.27563357133406	18.59792785829479	0.08814150452954
C	1.31210627968654	17.81600541717652	1.26197528373657
C	1.86526852988086	16.52866818941851	1.23417693156879
C	2.39879544975946	15.98578928119774	0.05796406864073
C	2.35719680614959	16.76770728336706	-1.10413123690620
C	1.80329012773949	18.05484028496335	-1.10217004093536

C	-1.66412366812576	24.56078174093253	-0.97036510311217
C	-2.20524945942039	25.84875443330489	-0.95121426607788
H	3.23674362147508	21.83688917811771	-1.80861064464104
H	5.70305542436889	21.51880904582421	-1.76370228572215
H	5.40283205306321	19.88166206615935	2.21896019076788
H	2.93737041933039	20.21322725116518	2.15824745448067
H	-2.38541407631393	19.42528340466058	2.03898794667958
H	-3.88269922745157	17.44020425783921	1.97187600459506
H	-2.56355413466581	16.60211531022891	-2.05147786802480
H	-1.07547715148966	18.59524551399329	-1.96828837313334
H	-0.40111529606667	24.44593087085040	2.18450747543182
H	-1.35922638796644	26.74089424159502	2.22761500953712
H	3.84231809514837	25.71103394917274	2.30911440918629
H	3.30397666388686	26.16567474191698	-1.93554931663287
H	-5.53753316641454	22.39573644519814	2.23566861789626
H	-5.60977273725352	21.79512227738789	-2.02436584552310
H	1.89527640807068	15.93724127921778	2.15354993416620
H	2.76042441976354	16.35813521186175	-2.03474833949910
H	-1.74395503610517	23.94360843948244	-1.86651454919785
H	-2.71261863832588	26.23476408843042	-1.83874087073800
H	-2.51833946584748	27.64695254608404	0.21064711271390
H	-3.98479216186301	16.02379829272140	-0.08203396989446
H	6.79819239285296	20.53981111161544	0.25611618751907
C	-7.16198014507353	22.27066119738173	0.10819080359916
C	-8.36929301428147	22.40191243787634	0.11105262846999
C	-2.98060878161099	21.37883093881565	-2.41012477406090
C	-2.88311714587956	22.20368220305280	2.61837320654399
C	2.30863239332380	23.53102128151678	2.67110805044225
C	1.58267080162254	24.14006925071158	-2.33640217097280
C	1.80482165547137	18.84332143739553	-2.39139309758484
C	0.74991955614853	18.33213125521911	2.56653692442582
H	-9.43683313819472	22.51774888743007	0.11376293281705
H	-3.50307678948906	21.86039770688390	-3.25105755325118
H	-1.93093023201011	21.70648773618491	-2.42618599675693

H	-2.98469100271113	20.29173481005073	-2.59220187639050
H	-1.92531133732391	21.66297333334274	2.62627899942055
H	-2.65908245527626	23.26471328642858	2.81467954771809
H	-3.49353516604867	21.83134710731637	3.45604595228350
H	3.06706103125779	22.74329296214903	2.80592576017791
H	2.38127558997878	24.21630663166247	3.52989627602169
H	1.32393763201710	23.04196168821307	2.70552849348525
H	2.26129174423929	24.34188846710558	-3.17947486592322
H	1.30874473799864	23.07513593098503	-2.36485265683409
H	0.65975328155776	24.71829054917331	-2.50510581950522
H	1.63999853597851	18.18373843249509	-3.25797158717254
H	1.02763814908146	19.62275539963934	-2.40601178673223
H	2.77278370615834	19.34948826268138	-2.53743849727159
H	0.72391284989606	19.43169107988288	2.60422527232240
H	-0.28406763497905	17.97946872972398	2.71162462731886
H	1.34593759066822	17.97678748552218	3.42162509084244
C	4.55612714076350	27.19835513882591	0.19652667205898
C	5.30483322161447	28.15503215028081	0.20281537336058
H	5.96692191170645	29.00002780871401	0.20818768065189
B	-3.01755828090107	6.34435433686890	0.02775915948705
N	-2.40359138454840	5.03881692488941	0.00027327432146
B	-0.97016324442100	4.86233293003049	-0.01640691948050
N	-0.14785691687463	6.04869661423892	-0.01491655845563
N	-2.14685847363887	7.49624205654280	0.04530871866688
C	1.27958929386529	5.90219531551963	-0.04834997945780
C	-2.73070184796553	8.80684441181615	0.09349205468140
C	-3.24673032037781	3.87604973625353	-0.01009911988202
C	1.96414905327190	5.37510553589778	1.05103925392440
C	3.35353935670985	5.23897555339858	1.02039549208867
C	4.07213003205955	5.62877113999394	-0.11408092073142
C	3.39190178172780	6.15757022433299	-1.21448382828876
C	2.00144559351882	6.29077546759828	-1.18229271021963
C	-3.43349787064993	9.31092671415954	-1.00673260779814
C	-3.99883762358397	10.58794562874682	-0.95781440804234

C	-3.86141823406594	11.37382309026163	0.18913273487575
C	-3.16189228409345	10.87069279393778	1.29171426020997
C	-2.59988222954844	9.59398332558403	1.24034108402301
C	-3.32589508544711	3.07042064001508	-1.15126743910817
C	-4.14471162040344	1.93766950655458	-1.16160400063949
C	-4.89603077405721	1.60377052300647	-0.03138140530902
C	-0.32105591217203	3.41710269536984	-0.03289682381308
C	0.15758501281392	2.85532213354072	-1.23672796470607
C	0.72774230092219	1.57993514870806	-1.23934235635322
C	0.85431802057614	0.83950918564637	-0.05210273159255
C	0.38340246638401	1.40490539178717	1.14463628716368
C	-0.20539771992800	2.67157789136206	1.16107931523631
C	-4.59354212901360	6.51034187906035	0.03195801245338
C	-5.32633186281292	6.40833976868857	-1.17122917042106
C	-6.71261980175593	6.57907886809120	-1.16625769314684
C	-7.40759666945914	6.83688540576878	0.02747367684879
C	-6.67593085335564	6.92692770978737	1.22311509026729
C	-5.28691696492886	6.77552348345509	1.23270385456103
B	-0.70710411026643	7.38096092996348	0.01855175475791
C	0.22206809888725	8.66587240472889	0.02284379653053
C	0.42383806708269	9.41194235195323	-1.15763374940570
C	1.24582027915383	10.54690959245856	-1.14210068344259
C	1.89134575646999	10.96888405402019	0.02756988318912
C	1.69004270883684	10.22011089924064	1.19463280362967
C	0.86703973511918	9.08571160699625	1.20530204890230
C	-3.99735801745920	3.53673544173653	1.11911347930217
C	-4.81726468937244	2.40640001960281	1.11173152037839
H	1.40066324562530	5.07013772471507	1.93463399482604
H	3.87554170539886	4.82862061665441	1.88738944935663
H	3.94351569266208	6.46831104498076	-2.10427780996518
H	1.46654057792496	6.69628716328817	-2.04222011003448
H	-3.52935305910019	8.70044888608998	-1.90544197323880
H	-4.54730837040787	10.96841051689555	-1.82241780704825
H	-3.05425632996991	11.47413949905727	2.19541416922936

H	-2.04727353473407	9.20027073818232	2.09682004898664
H	-2.74604311047559	3.33781440102492	-2.03580345805621
H	-4.19512789945108	1.31595169091451	-2.05778069303258
H	1.08926779341770	1.14590764112900	-2.17522014620577
H	0.48080558901040	0.83670179251209	2.07311681583805
H	-7.27442167708202	6.51056232351623	-2.10160096889282
H	-7.20920104134445	7.12529082646560	2.15660632941817
H	1.39710516322979	11.11263405354694	-2.06604925510907
H	2.17911697882243	10.53685819038214	2.12002966038782
H	-3.93876199173562	4.16738387407303	2.00866466328238
H	-5.39565095765730	2.15119282539036	2.00211166147987
H	-5.54206046299821	0.72421314535520	-0.04089965067158
H	-4.29481188608853	12.37518990109463	0.22527947493994
H	5.15825857656451	5.51840888727153	-0.14077132066941
C	-8.82823712902015	7.00540842700050	0.02429883599593
C	-10.03513693216231	7.14945291880276	0.02071635740356
C	-4.55548969607343	6.93473126872827	2.54423658592793
C	-4.64429706181314	6.09322803818669	-2.48153615588324
C	0.09669403987500	3.61888619778432	-2.53807170145279
C	-0.73088337979237	3.20346972563847	2.47340445154162
C	0.70618433062719	8.32264852375134	2.49992826387480
C	-0.24454018764649	9.02265087784989	-2.45591437902411
H	-11.10059249351240	7.27762525875129	0.01739048977085
H	-5.16534063681773	6.56433285170774	3.38325298051081
H	-3.59549877371437	6.39796105766267	2.55548298251029
H	-4.33527905270281	7.99742641126060	2.73479079836632
H	-3.59758521686716	6.43012267402258	-2.49874885071760
H	-4.63911179705155	5.00526442510571	-2.65985036272487
H	-5.17049146027004	6.56692480649186	-3.32449909093157
H	1.03167732402025	4.18014495214217	-2.69777290221307
H	-0.03247599313710	2.93499818001168	-3.39146800113636
H	-0.72636076758461	4.34830245847079	-2.55618171883381
H	-0.12192224946036	2.84283566605071	3.31689950594984
H	-0.74179367117326	4.30291064124544	2.50421003566874

H	-1.76586927162990	2.86338597055706	2.63927506765717
H	0.69905454843136	9.00647454781715	3.36312194458806
H	-0.22357609238586	7.73412736399869	2.52511499610322
H	1.53960877909675	7.61595194235088	2.64138064947200
H	-0.51383503631302	7.95593902246236	-2.48409921224356
H	-1.17504736612935	9.59526611292612	-2.60168694659884
H	0.41157079241053	9.22970366027588	-3.31583970972991
C	1.45261501731344	-0.46185889220731	-0.06017353126211
C	1.96045046952059	-1.56303389241204	-0.06499030761703
C	2.76610059689554	12.18391965651313	0.03148916181162
H	2.41054697056329	-2.53883411282821	-0.06897195136785
C	4.99525601526599	13.19156825495958	0.03354740471478
C	4.16477716221868	12.05978500287602	0.02766082666401
C	3.00754472864508	14.61852339907509	0.04602965326678
C	4.40269233499081	14.46434141214257	0.04252155075479
C	2.20085383570339	13.46944301509815	0.04076282480712
H	4.61484426482605	11.06272864802581	0.02116072239858
H	5.04060620628843	15.35261218877234	0.04725946713517
H	1.11246686854118	13.57715342510666	0.04448228856280
B	13.14610640213682	13.97484019226312	-0.02259755892269
N	13.79719420221458	12.68640856760191	-0.00146447509518
B	13.05300952726534	11.44868748319156	0.01764249133089
N	11.61225728062285	11.53116333273693	0.02910429679495
N	11.70245530117793	13.99794440526419	-0.02804518012215
C	10.84846539841288	10.31614759915580	0.06906415852964
C	11.02957020676513	15.26495618416590	-0.06599095052563
C	15.23147728049765	12.63546532621991	0.00207970545192
C	10.82967178414512	9.45416051756371	-1.03099096463915
C	10.08248101090300	8.27513451929670	-0.99367768825011
C	9.34685617509260	7.94609550560024	0.15013126786343
C	9.36158944160939	8.80738475168968	1.25101852708827
C	10.11131129065845	9.98537397204702	1.21089859682645
C	11.06955609616864	16.12281719998352	1.03846844196122
C	10.40928954972756	17.35382403500616	1.00059548058756

C	9.69998242764985	17.73575410965750	-0.14138525483694
C	9.66018271556950	16.88117436801524	-1.24744825119498
C	10.32232282492616	15.65271264603683	-1.20831936195488
C	15.92132125399051	12.20516789993817	1.14082055071768
C	17.31767782946866	12.15518285421227	1.14544070907626
C	18.03779102345452	12.53849110409546	0.01051134124560
C	13.79267432023091	10.04728086393025	0.02288387551455
C	13.95058676652437	9.31921416697316	1.22223062416071
C	14.60166822282926	8.08303967828544	1.21352272497403
C	15.09484356497349	7.53099766946147	0.01956700025628
C	14.93027104621147	8.25616649257550	-1.17265536931263
C	14.29719336956513	9.50127394514022	-1.17819368993786
C	13.98423909663389	15.31903794702423	-0.03427813120546
C	14.52672810873742	15.83547141374394	1.16313944190774
C	15.24236335785508	17.03505809535224	1.15056739401860
C	15.45271004032907	17.74203035901376	-0.04537803219239
C	14.92110303354526	17.21895246875080	-1.23578987839463
C	14.18886890360694	16.02899127189872	-1.23745590194221
B	10.90691201803907	12.79193281271909	0.00524514310022
C	9.32285210727802	12.86177566660479	0.01767832471852
C	8.62859683885210	13.17664036783036	1.20413834767517
C	7.23033090783515	13.27369226979637	1.19763878624839
C	6.48580815925721	13.05640748568988	0.03122420090710
C	7.18162478838806	12.72879276655493	-1.14045669701893
C	8.57947675167883	12.63348396347232	-1.16020832714898
C	15.95638856780332	13.01718546460187	-1.13196574751552
C	17.35215509487164	12.96868403789470	-1.12915122114095
H	11.40662724800113	9.71166902180587	-1.92168197441092
H	10.07378497749478	7.61233199503977	-1.86139060743528
H	8.78778228765851	8.56235422568669	2.14762505840195
H	10.13115823630562	10.65600161780208	2.07187998579847
H	11.61715505411358	15.81876964019764	1.93132566266996
H	10.45006919858883	18.01475301134237	1.86928690981323
H	9.11195259728487	17.17178958964053	-2.14676819311753

H	10.29136916769017	14.98158264845062	-2.06886854627756
H	15.35755970988611	11.91259716459791	2.02780161619571
H	17.84313490822747	11.81625696708682	2.04080803776953
H	14.72836369666190	7.52671614962381	2.14591522555447
H	15.30912946681462	7.83283980925103	-2.10643633624948
H	15.65062851048921	17.43665171402095	2.08174916029723
H	15.08219346678208	17.76130761923284	-2.17099271942509
H	6.70495080457965	13.51915164351192	2.12526725627596
H	6.61903267221073	12.56112714248668	-2.06299899333306
H	15.41938255040421	13.35506275236487	-2.01985596303562
H	17.90523707727984	13.26754529487195	-2.02243347593193
H	19.12922413245194	12.50315347939649	0.01445185174075
H	9.17947704200554	18.69521619917736	-0.17041199674797
H	8.76827462594503	7.02160411430689	0.18312993865072
C	16.19049067200059	18.96812852469014	-0.04973735716559
C	16.81598950042238	20.00930158432335	-0.05249569187842
C	13.60360899818257	15.54762506016504	-2.54388698363098
C	14.37132580841428	15.10500537511921	2.47588270519461
C	13.40026269215775	9.83317384322407	2.53119982090733
C	14.19113858575943	10.24900089186817	-2.48601054591461
C	9.26202422342637	12.27095884292122	-2.45891218988492
C	9.36103595228981	13.43346881770644	2.50060846056349
H	17.36830274959053	20.93011417498878	-0.05491336017432
H	14.27057493839196	15.78765443231394	-3.38665157408689
H	13.42480529370089	14.46237358973827	-2.54684503022307
H	12.63516568994064	16.03726429294878	-2.73431703311811
H	13.47787709143032	14.46383517017431	2.49411957388926
H	15.24233754079012	14.45474859803929	2.65930418102842
H	14.30172909271213	15.81357218074397	3.31576184397936
H	12.38501450783273	9.43894480137582	2.70172183954746
H	14.02915817932900	9.51338551343580	3.37656725448777
H	13.32976251945914	10.93026160595135	2.55239337741606
H	14.10012581836900	9.55121529599648	-3.33299190015658
H	13.32760604728712	10.93009890625532	-2.50921562687053

H	15.09223024019265	10.86087240987018	-2.65325593847195
H	8.71507828058906	12.68224374935156	-3.32159305800452
H	10.29719740746872	12.64217042038907	-2.50409884973902
H	9.30670568235443	11.17684434401907	-2.58286431887090
H	10.37889838437937	13.01550190039360	2.49659701624291
H	9.45464673811673	14.51545245672154	2.68860871542317
H	8.81814499421453	12.99449580008710	3.35324075367965
C	15.74770458663306	6.25783450919801	0.01685683824058
C	16.30064571826544	5.17577651788081	0.01406932562144
H	16.78912173902351	4.22004909512648	0.01162320169442.

S5 Characterization of the nanoporous covalent network

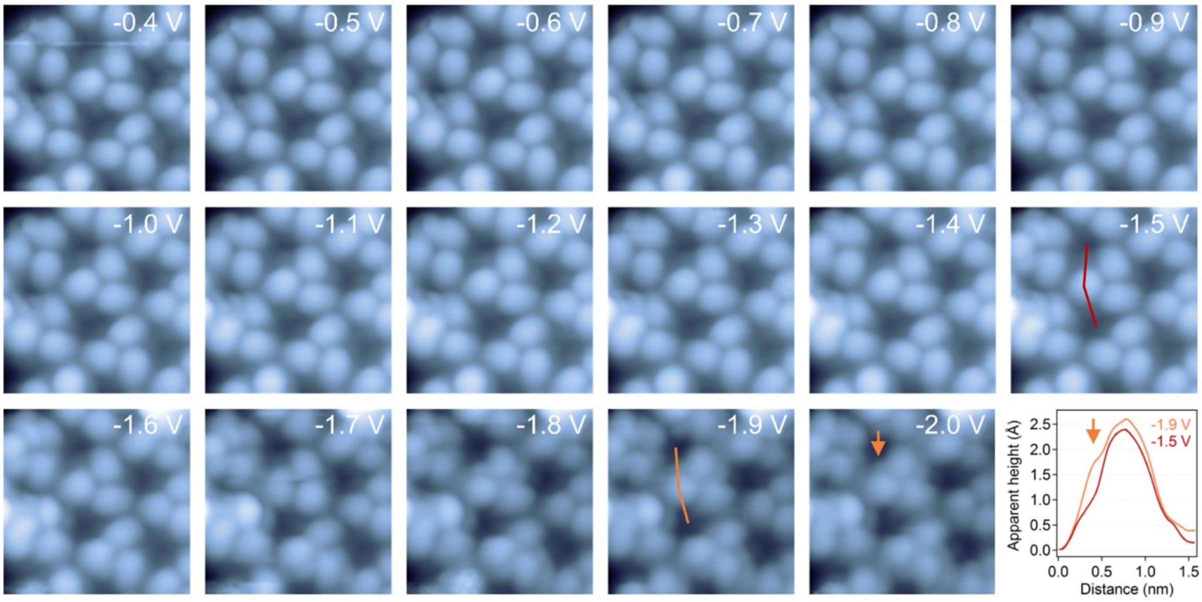

Figure S10. STM images of the hexagonal network at the indicated biases and a current setpoint of 10 pA (sizes: 3.54 nm x 3.64 nm). The panel on the bottom right compares apparent height profiles recorded at -1.5 V and -1.9 V, respectively. The selected path is indicated in the two STM images. The arrows mark features related to the phenyl groups. The increase in apparent height is consistent with the STS data presented in the main text.

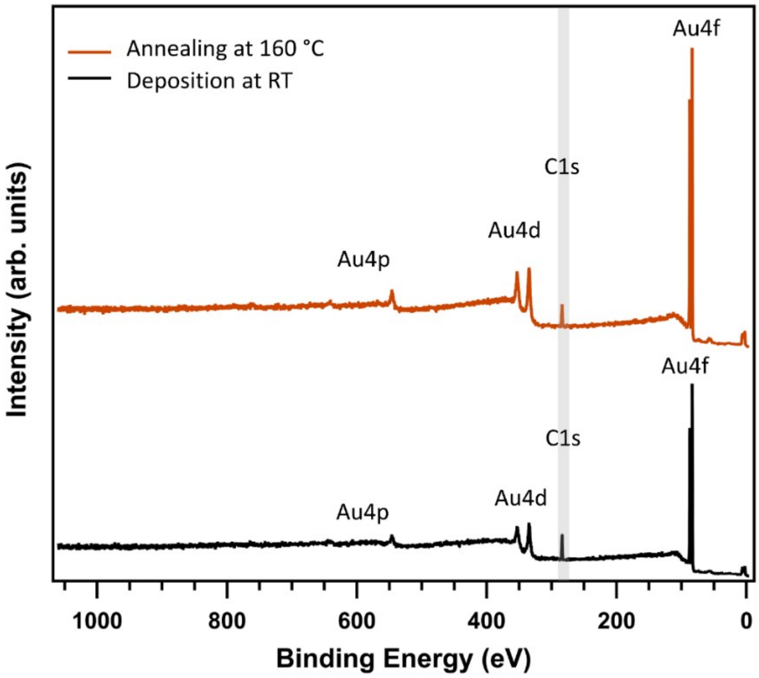

Figure S11. Wide scan XPS spectra of alkyne-terminated borazine on Au(111) collected after deposition at room temperature (black) and after annealing at 160 °C (red).

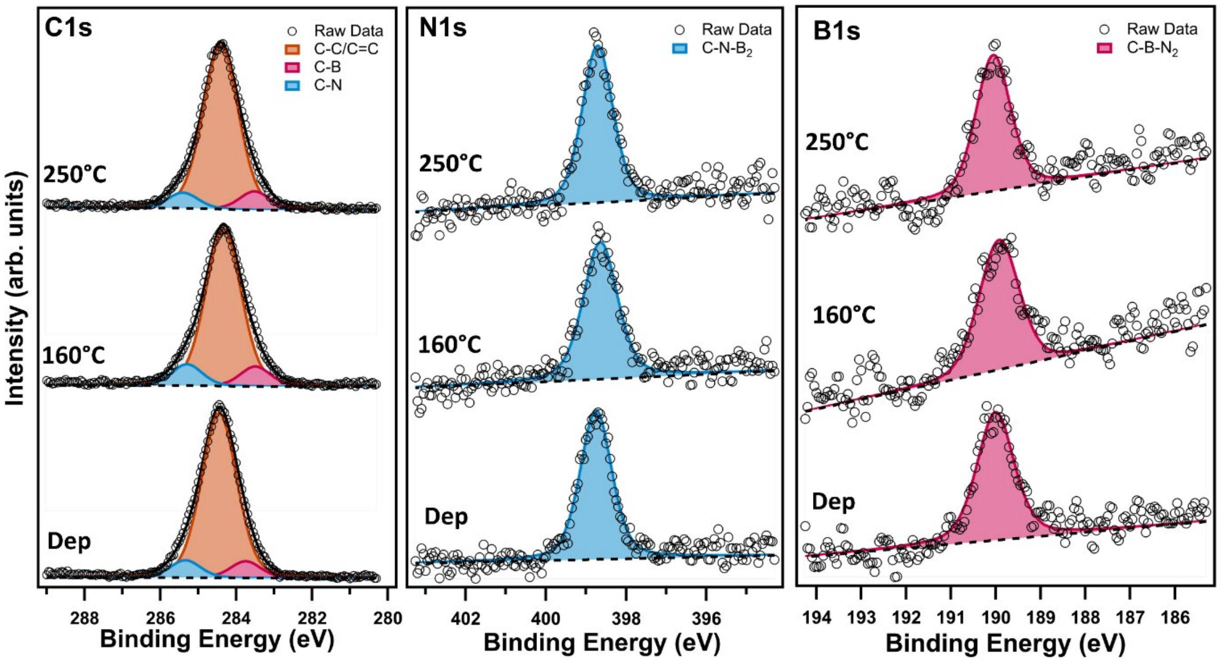

Figure S12. XPS spectra of the C1*s* (left panel), N1*s* (middle panel), B1*s* (right panel) core level regions of the alkyne-terminated borazine after deposition at room temperature, 160 °C and 250 °C. The corresponding fits are shown as well.

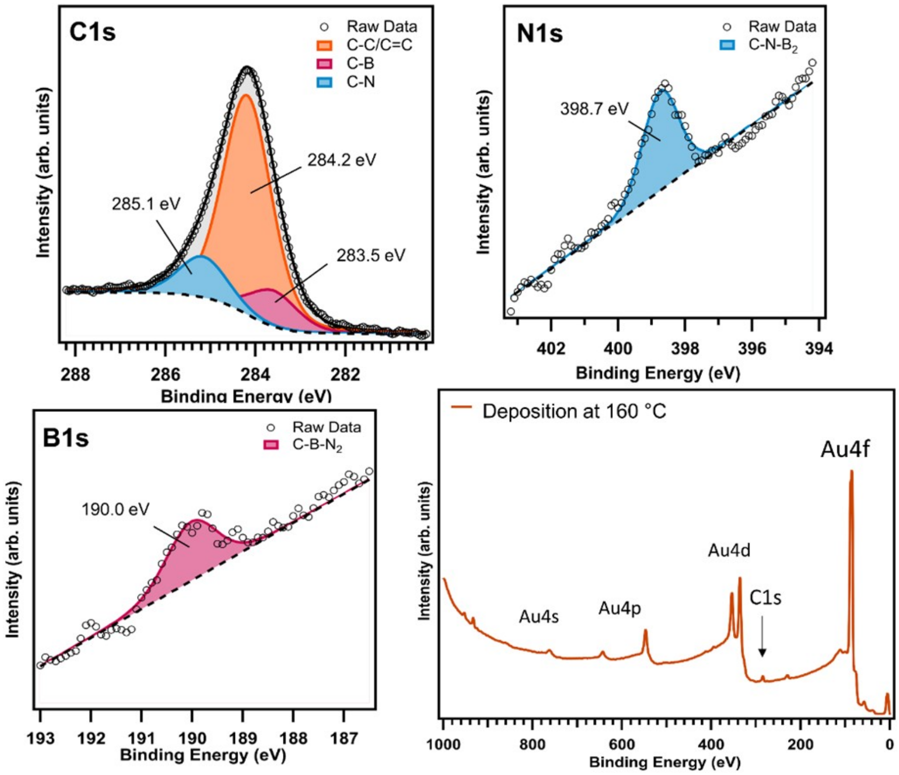

Figures S13. XPS spectra of the C1*s* (top left), N1*s* (top right), B1*s* (bottom left) core level regions and wide scan (bottom right) of a cyclotrimerized monolayer of the alkyne-terminated borazine after deposition at 160 °C. The corresponding fits are shown as well.

XPS experiments correlating to the STM measurements on the same sample (**Figure S13**) were conducted in a different UHV chamber equipped with a non-monochromated X-ray source (Mg Kα, hv = 1253.64 eV) and a SPECS PHOIBOS 100 hemispherical analyser. The sample was transferred from the STM to the XPS setup using a Ferrovac VSN40S UHV vacuum suitcase. The XPS measurement geometry was a magic angle with normal emission.

Table S1. Elemental composition in atomic percentages (at.%) of carbon (C), nitrogen (N) and boron (B) after deposition, annealing at 160 °C and 250 °C.

Atomic percentages (at.%)	C	N	B
**Annealing at 250 °C**	87.8 ± 0.4	7.3±3.4	5.0 ± 2.9
**Annealing at 160 °C**	86.3 ± 0.3	7.1±0.9	6.6 ± 2.5
**Deposition**	87.8 ± 0.5	6.7 ± 1.1	5.5 ± 2.1
**Theoretical**	88.8	5.6	5.6

The C1*s*, N1*s* and B1*s* core level spectra were measured in different spots to calculate the area, which after the appropriate normalization (photoemission cross section, transmission of the analyser) gives the atomic percentage with the uncertainty.

S6 References

1 D. Marinelli, F. Fasano, B. Najjari, N. Demitri and D. Bonifazi, *J. Am. Chem. Soc.*, 2017, **139**, 5503–5519.

2 F. Neese, *WIREs Computational Molecular Science*, 2022, **12**, e1606.
